# The association of *APOE* ε4 with cognitive function over the adult life course and incidence of dementia: 20 years follow-up of the Whitehall II study

**DOI:** 10.1186/s13195-020-00740-0

**Published:** 2021-01-04

**Authors:** Amin Gharbi-Meliani, Aline Dugravot, Séverine Sabia, Melina Regy, Aurore Fayosse, Alexis Schnitzler, Mika Kivimäki, Archana Singh-Manoux, Julien Dumurgier

**Affiliations:** 1 Université de Paris, Inserm U1153, Epidemiology of Ageing and Neurodegenerative Diseases, Paris, France; 2 Department of Epidemiology and Public Health, University College London, London, UK; 3 Cognitive Neurology Center, Lariboisiere - Fernand Widal Hospital, AP-HP, Université de Paris, 200 rue du Faubourg Saint Denis, 75010 Paris, France

**Keywords:** Apolipoprotein E, Cognitive aging, Cohort study, Dementia, Alzheimer’s disease

## Abstract

**Background:**

Approximately 25% of the general population carries at least one ε4 allele of the Apolipoprotein E (*APOE* ε4), the strongest genetic risk factor for late onset Alzheimer’s disease. Beyond its association with late-onset dementia, the association between *APOE* ε4 and change in cognition over the adult life course remains uncertain. This study aims to examine whether the association between Apolipoprotein E (*APOE*) ε4 zygosity and cognition function is modified between midlife and old age.

**Methods:**

A cohort study of 5561 participants (mean age 55.5 (SD = 5.9) years, 27.1% women) with *APOE* genotyping and repeated cognitive tests for reasoning, memory, and semantic and phonemic fluency, during a mean (SD) follow-up of 20.2 (2.8) years (the Whitehall II study). We used joint models to examine the association of *APOE* genotype with cognitive function trajectories between 45 and 85 years taking drop-out, dementia, and death into account and Fine and Gray models to examine associations with dementia.

**Results:**

Compared to non-carriers, heterozygote (prevalence 25%) and homozygote (prevalence 2%) *APOE* ε4 carriers had increased risk of dementia, sub-distribution hazard ratios 2.19 (95% CI 1.73, 2.77) and 5.97 (95% CI 3.85, 9.28) respectively. Using data spanning 45–85 years with non-ε4 carriers as the reference, ε4 homozygotes had poorer global cognitive score starting from 65 years; ε4 heterozygotes had better scores between 45 and 55 years, then no difference until poorer cognitive scores from 75 years onwards. In analysis of individual cognitive tests, better cognitive performance in the younger ε4 heterozygotes was primarily attributable to executive function.

**Conclusions:**

Both heterozygous and homozygous ε4 carriers had poorer cognition and greater risk of dementia at older ages. Our findings show some support for a complex antagonist pleiotropic effect of *APOE* ε4 heterozygosity over the adult life course, characterized by cognitive advantage in midlife.

**Supplementary Information:**

The online version contains supplementary material available at 10.1186/s13195-020-00740-0.

## Background

The ε4 allele of the Apolipoprotein E (*APOE*) gene is the strongest genetic risk factor for late onset Alzheimer’s disease (AD) [1]. Around 25% of the Caucasian population carries at least one ε4 allele [2], with a 3-fold increased risk of AD for heterozygotes and a nearly 15-fold increased risk for homozygotes compared to the ε3 homozygotes, the most common genotype [3]. *APOE* ε2 is less common and appears to have a protective effect on AD [4]. The mechanisms underlying the relationship between *APOE* ε4 and AD are thought to be complex [5], involving, e.g., β-amyloid (Aβ) peptide clearance [6], neuronal death [7], and phosphorylation of tau [8].

In addition to AD, *APOE* ε4 plays a role in other causes of dementia, including vascular dementia [9], and Lewy Body disease [10]. Although case-control and longitudinal studies have examined the association of *APOE* with dementia, its association with cognitive decline over the adult life course remains debated [11, 12]. Some studies show accelerated cognitive decline in *APOE ε4* homozygotes but not heterozygotes [13–15]. Furthermore, the association between *APOE* ε4 and cognition is thought to be modified by age; some [16–18] but not all studies [19, 20] report better cognitive performance among ε4 carriers at younger ages. The antagonistic pleiotropy hypothesis [21, 22], whereby a gene is thought to have different effects on health during different life stages, is a possible explanation for the age-varying association of *APOE* ε4 with cognitive performance over the life course [18, 22, 23]. However, much of the research on *APOE* is based on adults older than 65 years, followed for less than 10 years, making it difficult to ascertain how *APOE* shapes cognitive performance over the life course.

To address some of these limitations, we examined the relationship of homozygotes and heterozygotes *APOE* ε4 with cognitive decline from midlife to old age and incident dementia. The analysis of dementia takes competing risk of death into account and that for cognitive decline takes mortality, dementia, and drop-out into account using joint models.

## Methods

### Study population

The Whitehall II Study is an ongoing cohort study of persons originally employed by the British Civil Service, full details of which have been reported previously [24]. A total of 10,308 persons aged 35–55 years (67% male) were recruited to the study between 1985 and 1988 and have undergone clinical examination every 4 to 5 years. The baseline of the present study is 1997–1999 when a cognitive test battery was added to the protocol and repeated in 2002–2004, 2007–2009, 2012–2013, and 2015–2017.

### Cognitive function

The cognitive test battery, administered 5 times between 1997–1999 and 2015–2017, which consisted of 4 tests. Memory: participants were presented with a 20-word list of one or two syllable words at two second intervals, with 2 min time to write down as many words as they can recall, regardless of word order. Reasoning: participants had 10 min to complete the AH4-I (Alice Heim 4-I), a series of 65 verbal and mathematical reasoning items of increasing difficulty [25]. Verbal fluency: phonemic fluency was assessed via “S” words and semantic fluency via “animal” words tests. One minute was allowed for each test. To allow comparison between tests, we standardized all raw test scores to *z*-scores (mean = 0, standard deviation [SD] = 1). These *z*-scores were summed and re-standardized to yield the global cognitive score, a method that minimizes measurement error [26].

### Dementia

Dementia diagnosis was derived from three comprehensive electronic health records through to March 2019 [27]: NHS Digital’s Hospital Episode Statistics (HES) and Mental Health Services Data (MHDS), which include clinical diagnoses recorded during routine clinical contact in inpatient, outpatient, and community care in the NHS, including memory clinics, and the mortality register. The following ICD-10 codes were used for diagnosis of all-cause dementia: F00x-F03x, F05.1, and G30x-G31.0.

### *APOE* genotyping

DNA was extracted from whole blood samples, drawn at the 1997–1999 clinical examination. Two TaqMan assays (Rs429358 and Rs7412, Assay-On-Demand, Applied Biosystems) were used and run on a 7900HT analyzer (Applied Biosystems) and genotypes indicated by the Sequence Detection Software version 2.0 (Applied Biosystems). Genotyping was repeated for 511 participants and error rates were found to be lower than 0.15% [28].

### Covariates

Sociodemographic variables included age at baseline (1997–1999 examination), sex, marital status (married/cohabiting vs others), socioeconomic status using employment grade (three categories: high, intermediate, and low representing income and status at work), and education (three categories: lower secondary school, higher secondary school, and university/higher university degree).

### Statistical analysis

The current analyses were based on Caucasians, with data on *APOE* genotype and at least one measure of cognitive function. Baseline characteristics are presented for the analytic sample, by *APOE* genotype, and according to the occurrence (yes/no) of dementia or death during the follow-up. Proportions were calculated for categorical variables, while means and standard deviations were computed for continuous variables. Comparisons between groups were assessed using a *χ*^2^ test or analysis of variance as appropriate.

*APOE* was modeled as a function of the number of ε4 alleles (0, 1, or 2) and in detailed categories with ε2, ε3, and ε4 alleles. We first examined the association between *APOE* genotypes and incident dementia using Fine and Gray models for sub-distribution hazard ratio (SHR), to take into account the competing risk of death [29]. Age was considered as the time scale and participants were censored at onset of dementia, death, or end of follow-up (March 31, 2019), whichever came first. The initial model was adjusted for age (as time scale) and birth cohort (using 5-year categories) and subsequently for sex, education, marital status, and occupation.

We analyzed the relationship between *APOE* genotypes and cognitive decline using linear mixed models with age as time scale (age, age^2^, and age^3^ to model non-linear change). These models were adjusted for sex and its interaction with time and birth cohort, and both intercept and slope were fitted as random effects with unstructured covariance matrix. We used a joint modeling approach with the *stjm* command in Stata to model jointly cognitive decline (with initial linear mixed model) and time to exit from the follow-up, set at the earliest date from drop-out, dementia, or death (with a flexible parametric model). This approach links sub-models by including shared random effects that allow for dependency between the longitudinal process and time to drop-out, dementia, or death. We then estimated marginal predictions to determine the difference in cognitive function between *APOE* ε4 carriers compared to non-carriers at different ages between 45 and 85 years. Analyses were performed for the global cognitive score and repeated for each of the 4 cognitive tests. In sensitivity analysis, we reran the joint model after excluding all cases of dementia to test the robustness of the association between *APOE* genotypes and cognitive decline.

Two-tailed values of *p* < 0.05 were considered statistically significant. Analyses were performed using Stata 15 (StataCorp LP, College Station, TX).

## Results

### Demographic characteristics

**Fig. 1:**
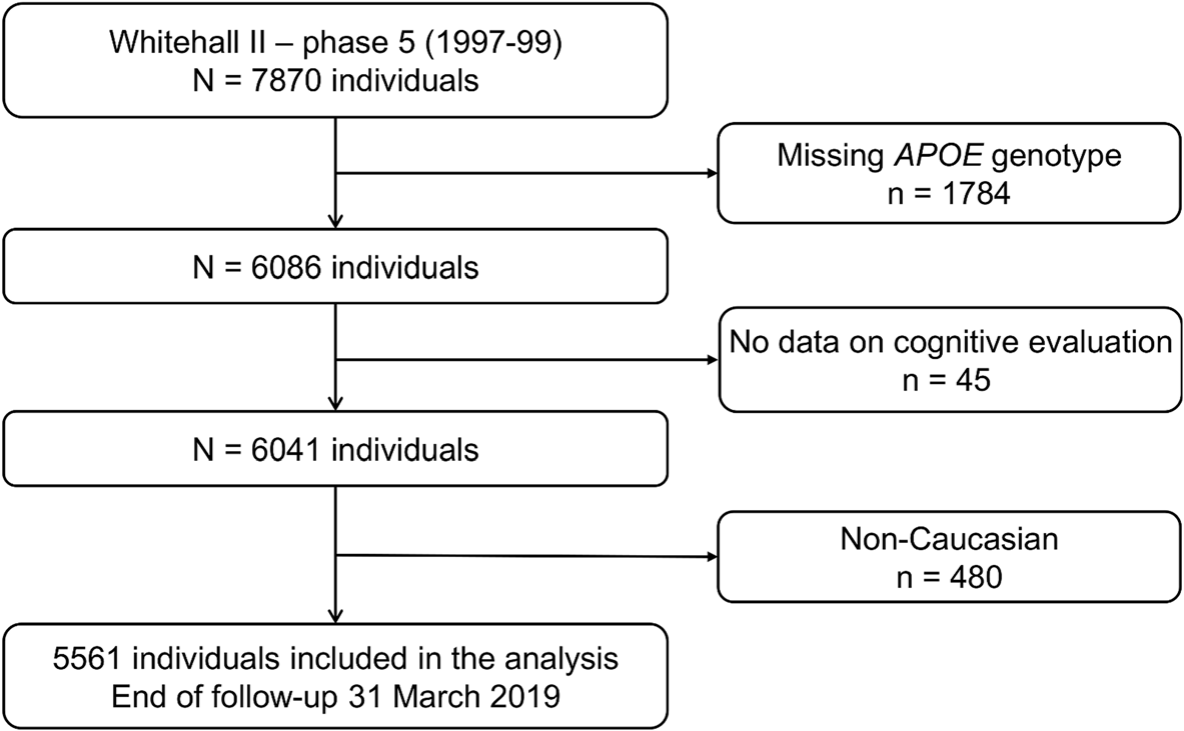
Flow chart of the study

A total of 7870 participants were included in the 1997–1999 clinical examination. Among them, 1784 were excluded from the present study due to missing data on *APOE* genotype and 45 for missing cognitive data. A further 480 participants were excluded as they were non-Caucasian; flow-chart of the study is presented in Fig. 1. A total of 5561 participants were included in the analysis, with a mean (SD) follow-up of 20.0 (2.8) years, corresponding to 111,132 person-years of follow-up.

Table 1 summarizes participants’ baseline characteristics, overall and by *APOE* genotype. Their mean (SD) age at start of the follow-up was 55.5 (5.9) years and 27% of them were women. The frequency of the alleles ε2, ε3, and ε4 was respectively 8%, 77%, and 15% in the study population. Fifty-nine percent of the study population were *APOE* ε3/ε3 homozygous, 27% carried at least one ε4 allele (heterozygotes 25%, homozygotes 2%), and 13% were either ε2/ε2 (0.6%) or ε2/ε3 (12.4%). No differences in term of socio-demographic characteristics were observed according to *APOE* genotype. Compared to ε3/ε3 participants (Additional file 1: Table S1), ε2/ε2 group had higher scores on memory (*p* = 0.035), phonemic fluency (*p* = 0.049), and semantic fluency (*p* = 0.049). The ε3/ε4 group also had higher scores on reasoning (*p* = 0.032) and phonemic fluency (*p* = 0.028) than ε3/ε3 homozygous. There was no difference in cognitive scores at baseline between the *APOE* ε4/ε4 and ε3/ε3 homozygotes.

**Table 1:** Baseline characteristics overall and by *APOE* genotype

Sample characteristics	***APOE*** genotype							***p*** value
	All	ε2ε2	ε2ε3	ε3ε3	ε2ε4	ε3ε4	ε4ε4	
	(***n*** = 5561)	(***n*** = 33)	(***n*** = 691)	(***n*** = 3296)	(***n*** = 139)	(***n*** = 1273)	(***n*** = 129)	
Age, years, mean (SD)	55.5 (5.9)	56.4 (5.6)	55.8 (6.2)	55.6 (5.9)	55.1 (5.9)	55.5 (6.0)	55.1 (6.0)	0.64
Women, *n* (%)	1508 (27.1)	10 (30.3)	175 (25.3)	930 (28.2)	33 (23.7)	327 (25.7)	33 (25.6)	0.35
Married/cohabiting, *n* (%)	4256 (76.5)	25 (75.8)	528 (76.4)	2524 (76.6)	103 (74.1)	977 (76.8)	99 (76.7)	0.99
Education level, *n* (%)								
Lower secondary school	2384 (42.9)	10 (30.3)	294 (42.6)	1423 (43.2)	47 (33.8)	565 (44.4)	45 (34.9)	
Higher secondary school	1524 (27.4)	12 (36.4)	191 (27.6)	920 (27.9)	42 (30.2)	324 (25.4)	35 (27.1)	
University degree or higher	1653 (29.7)	11 (33.3)	206 (29.8)	953 (28.9)	50 (36.0)	384 (30.2)	49 (38.0)	0.11
Occupation, *n* (%)								
Low	557 (10.0)	3 (9.1)	60 (8.7)	336 (10.2)	9 (6.5)	140 (11.0)	9 (7.0)	
Intermediate	2418 (43.5)	15 (45.5)	318 (46.0)	1449 (44.0)	70 (50.4)	514 (40.4)	52 (40.3)	
High	2586 (46.5)	15 (45.5)	313 (45.3)	1511 (45.8)	60 (43.2)	619 (48.6)	68 (52.7)	0.15
Follow time, years, mean (SD)	20.0 (2.8)	19.8 (3.1)	20.0 (2.8)	20.1 (2.7)	20.0 (2.5)	19.9 (2.9)	19.3 (3.4)	0.007
Cognitive function, mean (SD)								
Reasoning (range 0–65)	48.6 (9.7)	49.2 (10.1)	48.7 (9.5)	48.3 (9.8)	49.4 (9.2)	49.1 (9.4)	48.3 (10.3)	0.29
Memory (range 0–20)	7.1 (2.3)	8.1 (2.7)	6.8 (2.3)	7.1 (2.3)	6.9 (2.5)	7.1 (2.3)	6.9 (2.4)	0.010
Phonemic fluency (range 0–35)	17.3 (4.3)	18.8 (4.3)	17.2 (4.4)	17.2 (4.2)	17.1 (4.5)	17.5 (4.5)	16.5 (4.3)	0.03
Semantic fluency (range 0–35)	16.9 (4.0)	18.5 (4.0)	17.0 (4.2)	16.9 (4.0)	16.8 (3.6)	17.0 (4.0)	16.9 (4.0)	0.45
Standardized global cognitive score	− 0.0 (1.0)	0.4 (1.2)	− 0.0 (1.0)	− 0.0 (1.0)	− 0.0 (1.0)	0.1 (1.0)	− 0.1 (1.1)	0.10

### Association of *APOE* genotype and dementia

Table 2 presents baseline sample characteristics as a function of dementia and vital status over the follow-up. The 310 participants who developed dementia were older, were more often women, had a lower education level, had poorer cognitive performance, and were more likely to carry at least one *APOE* ε4 allele (46% vs 27%, *p* < 0.001). Seven hundred seventy-eight participants died during the follow-up. They were older, were more often single, and had a lower education level and poorer cognitive test scores.

**Table 2:** Baseline characteristics according to dementia and mortality status at the end of the follow-up

Sample characteristics	Dementia over follow-up			Mortality over follow-up		
	No	Yes	***p*** value	No	Yes	***p*** value
	(***n*** = 5251)	(***n*** = 310)		(***n*** = 4783)	(***n*** = 778)	
Age, years, mean (SD)	55.2 (5.8)	60.9 (5.0)	< 0.001	54.9 (5.7)	59.6 (5.8)	< 0.001
Women, *n* (%)	1403 (26.7)	105 (33.9)	0.006	1289 (27.0)	219 (28.2)	0.49
Married/cohabiting, *n* (%)	4032 (76.8)	224 (72.3)	0.07	3697 (77.3)	229 (71.9)	0.001
Education level, *n* (%)						
Lower secondary school	2209 (42.1)	175 (56.5)		2028 (42.4)	356 (45.8)	
Higher secondary school	1459 (27.8)	65 (21.0)		1301 (27.2)	223 (28.7)	
University degree or higher	1583 (30.2)	70 (22.6)	< 0.001	1454 (30.4)	199 (25.6)	0.02
Occupation, *n* (%)						
Low	493 (9.4)	64 (20.7)		449 (9.4)	108 (13.9)	
Intermediate	2297 (43.7)	121 (39.0)		2085 (43.6)	333 (42.8)	
High	2461 (46.9)	125 (40.3)	< 0.001	2249 (47.0)	337 (43.3)	< 0.001
Cognitive function, mean (SD)						
Reasoning	48.8 (9.5)	44.8 (11.1)	< 0.001	48.9 (9.5)	46.4 (10.5)	< 0.001
Memory	7.1 (2.3)	6.0 (2.2)	< 0.001	7.2 (2.3)	6.4 (2.3)	< 0.001
Phonemic fluency	17.3 (4.3)	15.8 (4.4)	< 0.001	17.4 (4.3)	16.3 (4.2)	< 0.001
Semantic fluency	17.0 (4.0)	15.2 (4.0)	< 0.001	17.1 (4.0)	16.2 (4.2)	< 0.001
Standardized global cognitive score	0.0 (1.0)	− 0.5 (1.0)	< 0.001	0.0 (1.0)	− 0.3 (1.0)	< 0.001
*APOE* genotype, *n* (%)						
ε2ε2	33 (0.6)	0 (0.0)		28 (0.6)	5 (0.6)	
ε2ε3	664 (12.7)	27 (8.7)		593 (12.4)	98 (12.6)	
ε3ε3	3156 (60.1)	140 (45.2)		2856 (59.7)	440 (56.6)	
ε2ε4	131 (2.5)	8 (2.6)		117 (2.5)	22 (2.8)	
ε3ε4	1162 (22.1)	111 (35.8)		1082 (22.6)	191 (24.6)	
ε4ε4	105 (2.0)	24 (7.7)	< 0.001	107 (2.2)	22 (2.8)	0.60

The association between *APOE* genotype and incident dementia, mean follow-up 20.0 (2.8) years, is presented in Table 3. Compared to non-ε4 carriers, the presence of ε4 allele was associated with an increased risk of dementia for both heterozygotes (SHR 2.19; 95% confidence interval 1.73 to 2.77) and homozygotes (5.97; 3.85 to 9.28), after adjustment for age and birth cohort. Further adjustment for sex, education, marital status, and occupation did not modify these associations.

**Table 3:** Fine and Gray sub-distribution hazard ratios (SHR) for incidence of dementia according to *APOE* genotype, taking into account the competing risk of death

***APOE*** genotype	***N*** (total)	% Dementia	Model 1^**a**^		Model 2^**b**^	
			SHR (95% CI)	***p*** value	SHR (95% CI)	***p*** value
Non-ε4 carrier	4020	4.1	1 (ref.)	–	1 (ref.)	–
ε4 heterozygote	1412	8.4	2.19 (1.73, 2.77)	< 0.001	2.22 (1.75, 2.81)	< 0.001
ε4 homozygote	129	18.6	5.97 (3.85, 9.28)	< 0.001	6.24 (3.99, 9.77)	< 0.001
ε2ε2/ε2ε3	724	3.7	0.81 (0.54, 1.21)	0.30	0.82 (0.54, 1.23)	0.33
ε3ε3	3296	4.2	1 (ref.)	–	1 (ref.)	–
ε2ε4	139	5.8	1.48 (0.73, 3.00)	0.27	1.51 (0.74, 3.07)	0.25
ε3ε4	1273	8.7	2.17 (1.69, 2.78)	< 0.001	2.21 (1.72, 2.83)	< 0.001
ε4ε4	129	18.6	5.75 (3.69, 8.98)	< 0.001	6.01 (3.82, 9.46)	< 0.001

^a^Adjusted for age (as time scale) and birth cohort in 5 categories

^b^Adjusted for age, birth cohort, sex, level of education, marital status, and occupation

### *APOE* genotype and cognitive function trajectories

A total of 0.4% participants dropped-out after the first wave of cognitive data collection, 9.1% after the second wave, 8.7% after the third wave, and 11.8% after the fourth wave; 69.9% of participants included in the analyses provided data at all waves. Participants with fewer follow-up examinations were more likely to be older, women, and less educated and had lower cognitive scores at baseline. *APOE* ε4 status was not associated with participation over the follow-up (Additional file 2: Table S2).

**Fig. 2:**
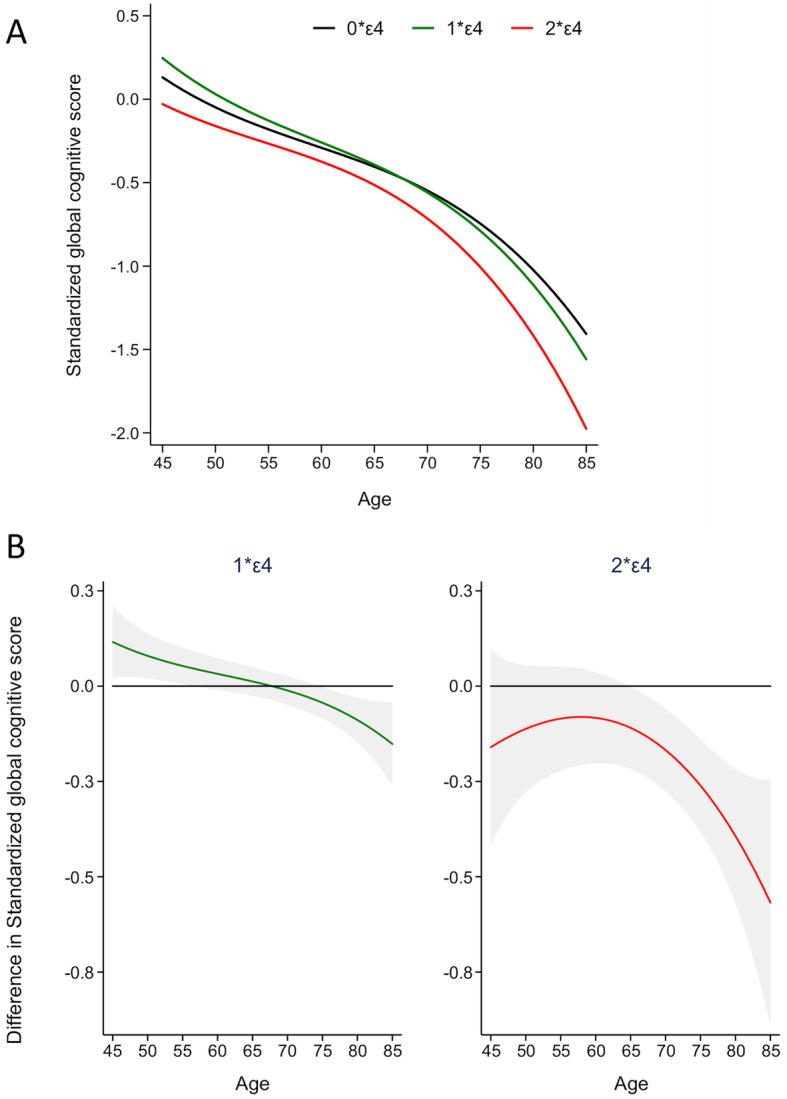
Global cognitive score over the adult life course as a function of number of *APOE* ε4 alleles. Analysis are undertaken using joint models, using age as time scale (age, age^2^, and age^3^), and adjusted for sex, marital status, education level, occupation, and their interactions with time. **a** Global cognitive score trajectories according to the number of *APOE* ε4 alleles. **b** Difference in global cognitive score in *APOE* ε4 homozygotes and heterozygotes compared to non-ε4 carriers. Gray shaded intervals represent 95% confidence intervals of the estimates

Trajectories of the global cognitive score between 45 and 85 years as a function of the number of *APOE* ε4 alleles (no- ε4, heterozygotes, and homozygotes) are presented in Fig. 2a. Overall, the global cognitive score declined with age in all the three groups (*p* < 0.001). Compared to non-ε4 carriers, ε4 homozygotes had poorer global cognitive score from 65 years onwards (Fig. 2b, Table 4). ε4 heterozygotes had better performances than non-ε4 carriers between 45 and 55 years, then no differences between 60 and 70 years, and poorer performance from 75 years onwards (Fig. 2b, Table 4). Further detailed analysis (Additional file 3: Table S3) showed the group (ε2/ε2, ε2/ε3) to have better cognitive performance after the age of 80 compared to ε3/ε3 (*p* = 0.04), while no differences were observed for ε2/ε4 individuals. In sensitivity analysis, we reran the joint models after exclusion of 208 participants with incident dementia over the follow-up and found similar results.

**Table 4:** Difference in cognitive score between ε4 heterozygotes and homozygotes compared to non-ε4 carriers at ages 45 to 85 years

	Difference in standardized global cognitive score		Difference in standardized memory score		Difference in standardized reasoning score		Difference in standardized semantic fluency score		Difference in standardized phonemic fluency score	
Age	β (95% CI)^**a**^	***p*** value	β (95% CI)^**a**^	***p*** value	β (95% CI)^**a**^	***p*** value	β (95% CI)^**a**^	***p*** value	β (95% CI)^**a**^	***p*** value
**ε4 heterozygotes vs non ε4 carriers**										
45	0.11 (0.02, 0.20)	0.01	0.02 (− 0.13, 0.17)	0.83	0.13 (0.03, 0.22)	0.01	− 0.00 (− 0.13, 0.13)	0.99	0.15 (0.02, 0.28)	0.02
50	0.08 (0.02, 0.13)	0.005	0.03 (− 0.05, 0.10)	0.44	0.09 (0.03, 0.14)	0.003	0.01 (− 0.06, 0.07)	0.84	0.10 (0.03, 0.17)	0.003
55	0.06 (0.01, 0.10)	0.02	0.03 (− 0.02, 0.09)	0.26	0.06 (0.01, 0.11)	0.02	0.01 (− 0.04, 0.06)	0.69	0.06 (0.00, 0.11)	0.04
60	0.03 (− 0.01, 0.08)	0.10	0.02 (− 0.02, 0.07)	0.33	0.04 (− 0.01, 0.09)	0.09	0.01 (− 0.04, 0.06)	0.70	0.02 (− 0.03, 0.07)	0.36
65	0.01 (− 0.02, 0.05)	0.47	0.00 (− 0.04, 0.05)	0.84	0.03 (− 0.02, 0.08)	0.31	0.00 (− 0.04, 0.05)	0.95	0.00 (− 0.04, 0.05)	0.89
70	− 0.01 (− 0.05, 0.03)	0.57	− 0.03 (− 0.08, 0.02)	0.23	0.00 (− 0.05, 0.05)	0.97	− 0.01 (− 0.06, 0.03)	0.54	− 0.00 (− 0.05, 0.05)	0.91
75	− 0.04 (− 0.08, − 0.00)	0.04	− 0.08 (− 0.14, − 0.02)	0.007	− 0.04 (− 0.10, 0.02)	0.19	− 0.04 (− 0.10, 0.01)	0.13	0.01 (− 0.05, 0.11)	0.84
80	− 0.08 (− 0.14, − 0.03)	0.002	− 0.15 (− 0.23, − 0.06)	0.001	− 0.10 (− 0.18, − 0.03)	0.006	− 0.08 (− 0.16, − 0.00)	0.04	0.03 (− 0.05, 0.11)	0.44
85	− 0.14 (− 0.24, − 0.04)	0.006	− 0.23 (− 0.40, − 0.07)	0.006	− 0.20 (− 0.33, − 0.07)	0.002	− 0.13 (− 0.28, 0.02)	0.09	0.08 (− 0.08, 0.23)	0.34
**ε4 homozygotes vs non ε4 carriers**										
45	− 0.17 (− 0.41, 0.08)	0.18	− 0.12 (− 0.52, 0.29)	0.56	− 0.06 (− 0.32, 0.20)	0.68	0.05 (− 0.29, 0.39)	0.78	− 0.29 (− 0.63, 0.06)	0.11
50	− 0.11 (− 0.27, 0.05)	0.18	− 0.05 (− 0.26, 0.15)	0.62	− 0.05 (− 0.21, 0.11)	0.52	0.01 (− 0.18, 0.19)	0.95	− 0.13 (− 0.32, 0.06)	0.17
55	− 0.08 (− 0.21, 0.05)	0.24	− 0.04 (− 0.21, 0.12)	0.59	− 0.06 (− 0.21, 0.09)	0.43	− 0.04 (− 0.19, 0.11)	0.63	− 0.04 (− 0.20, 0.12)	0.61
60	− 0.08 (− 0.19, 0.04)	0.19	− 0.08 (− 0.22, 0.06)	0.28	− 0.08 (− 0.22, 0.06)	0.27	− 0.09 (− 0.22, 0.05)	0.22	− 0.01 (− 0.15, 0.14)	0.91
65	− 0.11 (− 0.21, 0.00)	0.05	− 0.14 (− 0.27, − 0.01)	0.03	− 0.12 (− 0.27, 0.02)	0.09	− 0.14 (− 0.27, − 0.01)	0.03	− 0.02 (− 0.16, 0.12)	0.76
70	− 0.17 (− 0.27, − 0.06)	0.003	− 0.22 (− 0.36, − 0.07)	0.003	− 0.20 (− 0.35, − 0.04)	0.01	− 0.21 (− 0.35, − 0.07)	0.003	− 0.07 (− 0.22, 0.08)	0.33
75	− 0.25 (− 0.37, − 0.14)	< 0.001	− 0.29 (− 0.46, − 0.11)	0.001	− 0.31 (− 0.48, − 0.14)	< 0.001	− 0.30 (− 0.46, − 0.14)	< 0.001	− 0.16 (− 0.33, 0.01)	0.07
80	− 0.37 (− 0.54, − 0.21)	< 0.001	− 0.34 (− 0.60, − 0.08)	0.009	− 0.46 (− 0.68, − 0.24)	< 0.001	− 0.41 (− 0.64, − 0.18)	0.001	− 0.26 (− 0.50, − 0.02)	0.04
85	− 0.53 (− 0.82, − 0.23)	0.001	− 0.36 (− 0.86, 0.13)	0.15	− 0.66 (− 1.04, − 0.28)	0.001	− 0.54 (− 0.99, − 0.09)	0.019	− 0.37 (− 0.83, 0.10)	0.12

^a^Analysis undertaken using joint models (the linear mixed submodel using age as time scale (age, age^2^, and age^3^) is adjusted for sex, marital status, education level, and occupation and their interaction with time terms if significant)

**Fig. 3:**
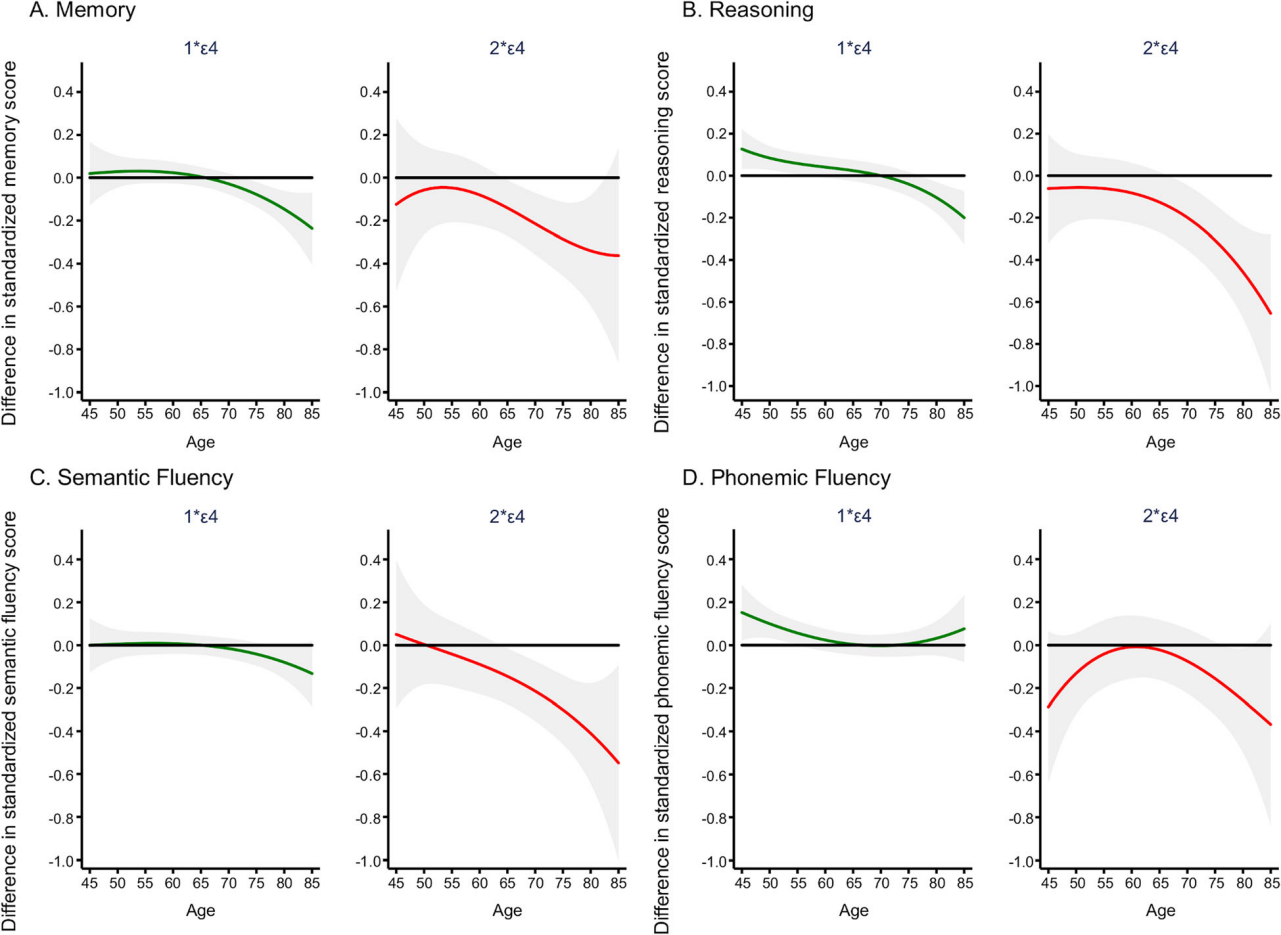
Difference in standardized cognitive tests of memory (**a**), reasoning (**b**), semantic (**c**), and phonemic fluency (**d**) in *APOE* ε4 heterozygotes and homozygotes compared to non-ε4 carriers. Analysis are undertaken using joint models, using age as time scale (age, age^2^, and age^3^), and adjusted for sex, marital status, education level, occupation, and their interactions with time. Gray-shaded intervals represent 95% confidence intervals of the estimates

Further analyses were undertaken using performance on individual cognitive tests between the ages of 45 and 85 years as the outcome; results are shown in Table 4 and Fig. 3. Participants who were ε4 heterozygous had better performance on reasoning and phonemic fluency than non-ε4 carriers at younger ages and poorer performance on memory, reasoning, and semantic fluency at older ages. For all cognitive tests, ε4 homozygotes showed lower cognitive performance at older ages.

## Discussion

This longitudinal study based on 5561 men and women presents two key findings. One, we confirmed that the ε4 allele of *APOE* is associated with accelerated cognitive decline over the adult life course, not only homozygotes but also heterozygotes, irrespective of dementia occurrence. Compared to non-ε4 carriers, worse cognitive performance among ε4 carriers was noticeable from 65 years of age for homozygotes and from 75 years for heterozygotes. Two, we found a seemingly paradoxical effect of *APOE* ε4 in heterozygotes who had better performance on the global cognitive score than non-ε4 carriers up to the age of 55 years. More fine grained analyses suggested that better cognitive performance in the younger ε4 heterozygotes was primarily in tests that tap into executive function (reasoning, phonemic fluency). These results taken together provide support for the antagonistic pleiotropic hypothesis as cognitive performance was better at younger ages in *APOE* ε4 heterozygotes and both heterozygous and homozygous *APOE* ε4 carriers also had higher risk of dementia at older ages. The strength of the associations with cognitive performance was comparable to that in previous studies which did not include dementia follow-up [30, 31].

Few previous studies have examined the association between *APOE* genotype and cognitive decline over the adult life course as most studies are based on older adults who were followed for cognitive outcomes for less than 10 years [13–15, 32]. Several studies did not distinguish between ε4 heterozygotes and homozygotes [32–34], and studies making this distinction did not find evidence of faster cognitive decline in ε4 heterozygous carriers [13–15]. In the Arizona *APOE* cohort (*n* = 815) with mean age of participants at baseline being 60.1 years and mean follow-up 5 years, ε4 homozygous had a more pronounced cognitive decline than ε4 non-carriers but no significant difference was observed for ε4 heterozygotes [14]. In another study on 621 participants (mean age 58 years, follow-up 6 years), a more pronounced decline was likewise observed only for ε4 homozygotes [13]. This was also the case in the MRC National Survey of Health and Development cohort study [15]. It is possible that the limited follow-up in these studies did not allow the age-dependent association between heterozygous *APOE* ε4 and cognitive function to be detected. Such information is important as ε4 homozygotes represent a small proportion of the population but the prevalence of ε4 heterozygotes is over 20%.

The mechanisms underlying the association between *APOE* ε4 and cognitive decline remain poorly understood; further research using AD biomarkers may provide insight into these mechanisms. Several studies have shown that APOE ε4 carriers in non-demented population have an increased incidence of beta-amyloid PET positivity compared to non-carriers [35]. A recent amyloid PET based study suggests that APOE ε4 carriers may reach abnormal level of neocortical Aβ-amyloid at the age of 63 compared to 78 years in non-carriers [36], suggesting a 15-year difference between these 2 categories. Accumulation of protein Tau is also likely to play a role as a study showed an increase of tau PET uptake in the entorhinal cortex and hippocampus among ε4-carriers independently of Aβ load [37]. Poorer cognition has been related to tau PET accumulation, even among Aβ-negative ε4 carriers [38], suggesting that the APOE ε4 allele may enhance the vulnerability to progressive tau accumulation in the AD spectrum [39].

To our knowledge, ours is the first study to show that ε4 allele heterozygosity may have a differential effect on cognition as a function of age. The long follow-up allowed us to show that compared to non ε4 carriers, ε4 heterozygotes had poorer cognitive scores after the age of 75 years old but better performance before the age of 55. Few cross-sectional or short longitudinal studies have been able to show better cognitive performance in young ε4 carriers [17, 34, 40]. An experimental study on mice found that ε4 allele was initially associated with better spatial memory in young animals and then deleterious effect at later ages [41]. Interestingly, we found that the early cognitive benefit associated with the ε4 allele is mainly in executive function (reasoning, phonemic fluency), while no difference was observed for memory or semantic tasks which involve temporal and temporal intern area. This is consistent with several metabolic PET imaging studies which have found that *APOE* ε4 allele in the normal population is associated with a decrease in metabolism in the posterior regions of the brain (parietal, posterior cingulate), but also with an increase of metabolism in the anterior frontal area [42, 43]. A recent meta-analysis of studies on the age range from 2 to 40 years did not find differences in cognitive performance between *APOE* ε4 carriers and non-carriers, with the authors concluding that there was no support for the antagonistic pleiotropic hypothesis [20]. As this meta-analysis combined *APOE* ε4 homozygotes and heterozygotes, the results are not directly comparable to our study. It is also possible that the effect we observed is not innate but acquired and may appear after the 4th decade of life in reaction of early biochemical processes involved in neurodegenerative diseases, like the onset of beta-amyloid deposition observed in the posterior area of the brain in AD pathology [44].

It is unclear why *APOE* ε4 has remained highly prevalent in the population over the course of evolution despite its deleterious effects on dementia and cardiovascular health [45]. Our results show that *APOE* ε4 could confer a cognitive advantage before the age of 55 years, especially in reasoning and psychomotor speed, which could have contributed to the preservation of this allele over the long course of premodern human history when mean life expectancy was lower than 50 years [46]. Another recent study also found that *APOE* ε4 carriers may particularly benefit of protective effect on the brain connectivity of the physical activity [47].

### Limitations

This study has several strengths, including its large sample size and the long follow-up. We also used appropriate statistical methods, i.e., joint modeling, to take into account the potential selection bias arising from mortality, dementia, and drop-out. Despite the long duration of follow-up, we were not able to model the relationship before the age of 45 years and thus examine whether the cognitive benefits related to *APOE* ε4 are evident earlier in the life course. A further limitation is that we were not able to completely rule out the role of AD/dementia, in particular preclinical dementia, in cognitive decline observed in APOE ε4 carriers. To limit this bias, we censored individuals at diagnosis of dementia in our primary analyses and then tested the robustness of our results by completely excluding participants diagnosed with dementia over the follow-up. The lack of preclinical markers of AD/dementia biomarkers is a limitation. Ongoing advances in plasma-based biomarkers will be an important opportunity in the future to better understand the mechanisms underlying these associations.

## Conclusions

In summary, our results show some support for a complex antagonist pleiotropic effect of *APOE* ε4 heterozygosity during adult life course and confirm that both heterozygous and homozygous ε4 carriers have poorer cognition at older ages. Further research using different population settings in similar life course studies is needed to test the generalizability of our findings.

## Supplementary Information

**Additional file 1: Table S1**. Baseline cognitive function as a function of *APOE* genotype with ε3ε3 as the reference.

**Additional file 2: Table S2.** Baseline characteristics of participants as a function of the number of waves of cognitive data over the follow-up.

**Additional file 3: Table S3.** Difference in Standardized Global Cognitive Score between 45 and 85 Years by *APOE* in 5 Categories.

## Data Availability

Bona fide researchers can apply to access Whitehall II data via the national dementia platform (https://www.dementiasplatform.uk/) or the study specific mechanism, details on https://www.ucl.ac.uk/epidemiology-health-care/research/epidemiology-and-public-health/research/whitehall-ii/data-sharing.

## Abbreviations

AD: Alzheimer’s disease
APOE: Apolipoprotein E
SD: Standard deviation
SHR: Sub-distribution hazard ratio
