# Supplementary material for: The association of APOE ε4 with cognitive function over the adult life course and incidence of dementia: 20 years follow-up of the Whitehall II study
Source: Alzheimers Res Ther. 2021 Jan 4;13:5. doi: 10.1186/s13195-020-00740-0 (PMC7784268; doi:10.1186/s13195-020-00740-0)
Supplement: Supplementary file 1 — Additional file 1: Table S1. Baseline cognitive function as a function of APOE genotype with ε3ε3 as the reference. [file 13195_2020_740_MOESM1_ESM.docx]

**Table S1**. Baseline cognitive function as a function of *APOE* genotype with ε3ε3 as the reference.

|  |  | Comparison to ε3ε3, β(SE), p-value | | | | |
| --- | --- | --- | --- | --- | --- | --- |
| Cognitive function | ε3ε3 | ε2ε2 | ε2ε3 | ε2ε4 | ε3ε4 | ε4ε4 |
| Reasoning | Ref. | 0.93 (1.90), 0.62 | 0.39 (0.45), 0.39 | 1.15 (0.94), 0.22 | 0.78 (0.36), 0.03 | 0.01 (0.96), 0.99 |
| Memory | Ref. | 0.97 (0.46), 0.04 | -0.33 (0.11), 0.003 | -0.18 (0.23), 0.42 | 0.00 (0.09), 0.99 | -0.19 (0.23), 0.40 |
| Phonemic fluency | Ref. | 1.66 (0.84), 0.05 | 0.05 (0.20), 0.81 | -0.13 (0.42), 0.76 | 0.34 (0.16), 0.03 | -0.71 (0.43), 0.10 |
| Semantic fluency | Ref. | 1.56 (0.79), 0.05 | 0.12 (0.19), 0.52 | -0.15 (0.39), 0.71 | 0.10 (0.15), 0.48 | -0.02 (0.40), 0.97 |
| Standardized Global Cognitive Score | Ref. | 0.43 (0.20), 0.03 | -0.02 (0.05), 0.67 | -0.01 (0.10), 0.93 | 0.06 (0.04), 0.09 | -0.08 (0.10), 0.39 |
