# Supplementary material for: The association of APOE ε4 with cognitive function over the adult life course and incidence of dementia: 20 years follow-up of the Whitehall II study
Source: Alzheimers Res Ther. 2021 Jan 4;13:5. doi: 10.1186/s13195-020-00740-0 (PMC7784268; doi:10.1186/s13195-020-00740-0)
Supplement: Supplementary file 2 — Additional file 2: Table S2. Baseline characteristics of participants as a function of the number of waves of cognitive data over the follow-up. [file 13195_2020_740_MOESM2_ESM.docx]

**Table S2.** Baseline characteristics of participants as a function of the number of waves of cognitive data over the follow-up.

|  |  | |  |  |  |  |  |
| --- | --- | --- | --- | --- | --- | --- | --- |
|  | Number of waves of cognitive data over the follow-up | | | | | | |
| Characterististics (baseline) | | 5 | 4 | 3 | 2 | 1 | p-value |
| N (% of the total sample) | | 3889 (69.9) | 658 (11.8) | 482 (8.7) | 508 (9.1) | 24 (0.4) |  |
| Age, years, mean (SD) | | 57.8 (6.0) | 57.6 (6.1) | 57.8 (6.3) | 57.8 (6.0) | 60.79 (4.49) | <0.001 |
| Women, n (%) | | 961 (24.7) | 198 (30.1) | 153 (31.7) | 181 (35.6) | 15 (62.5) | <0.001 |
| University degree or higher, n (%) | | 1294 (33.3) | 147 (22.3) | 100 (20.8) | 110 (21.7) | 2 (8.3) | <0.001 |
| Standardized Global cognitive score, mean (SD) | | 0.12 (0.96) | -0.24 (1.00) | -0.21 (0.99) | -0.46 (1.07) | -1.48 (1.06) | <0.001 |
| APOE e4 carriers, n (%) | | 1060 (27.3) | 175 (26.6) | 161 (33.4) | 139 (27.4) | 6 (25.0) | 0.07 |
|  |  | |  |  |  |  |  |

Percentages are presented in columns apart for the first line.
