# Supplementary material for: The association of APOE ε4 with cognitive function over the adult life course and incidence of dementia: 20 years follow-up of the Whitehall II study
Source: Alzheimers Res Ther. 2021 Jan 4;13:5. doi: 10.1186/s13195-020-00740-0 (PMC7784268; doi:10.1186/s13195-020-00740-0)
Supplement: Supplementary file 3 — Additional file 3: Table S3. Difference in Standardized Global Cognitive Score between 45 and 85 Years by APOE in 5 Categories. [file 13195_2020_740_MOESM3_ESM.docx]

**Table S3.** Difference in Standardized Global Cognitive Score between 45 and 85 Years by *APOE* in 5 Categories.

|  | **APOE in 5 categories^a^** | | | | | | | | |
| --- | --- | --- | --- | --- | --- | --- | --- | --- | --- |
|  | **ε2ε2/ε2ε3** | | **ε3ε3** | **ε2ε4** | | **ε3ε4** | | **ε4ε4** | |
| **Age** | **Difference  (95% CI)** | ***p-value*** |  | **Difference  (95% CI)** | ***p-value*** | **Difference  (95% CI)** | ***p-value*** | **Difference  (95% CI)** | ***p-value*** |
|  |  |  |  |  |  |  |  |  |  |
| **45** | 0.02 (-0.15, 0.10) | 0.72 | *Ref.* | -0.13 (-0.40, 0.14) | 0.34 | 0.14 (0.04, 0.24) | 0.007 | -0.17 (-0.43, 0.10) | 0.22 |
| **50** | 0.02 (-0.10, 0.06) | 0.61 | *Ref.* | -0.06 (-0.22, 0.10) | 0.48 | 0.09 (0.03, 0.15) | 0.004 | -0.12 (-0.28, 0.05) | 0.17 |
| **55** | -0.02 (-0.08, 0.04) | 0.56 | *Ref.* | -0.03 (-0.16, 0.10) | 0.67 | 0.06 (0.01, 0.11) | 0.02 | -0.09 (-0.23, 0.05) | 0.21 |
| **60** | -0.02 (-0.07, 0.04) | 0.58 | *Ref.* | -0.03 (-0.15, 0.08) | 0.59 | 0.04 (-0.01, 0.08) | 0.10 | -0.09 (-0.21, 0.03) | 0.16 |
| **65** | -0.01 (-0.06, 0.04) | 0.76 | *Ref.* | -0.05 (-0.15, 0.05) | 0.34 | 0.02 (-0.02, 0.06) | 0.40 | -0.11 (-0.22, -0.00) | 0.04 |
| **70** | 0.01 (-0.04, 0.06) | 0.73 | *Ref.* | -0.07 (-0.18, 0.04) | 0.20 | -0.00 (-0.04, 0.04) | 0.85 | -0.17 (-0.28, -0.06) | 0.003 |
| **75** | 0.04 (-0.02, 0.09) | 0.19 | *Ref.* | -0.07 (-0.19, 0.04) | 0.22 | -0.03 (-0.08, 0.01) | 0.14 | -0.25 (-0.38, -0.13) | < 0.001 |
| **80** | 0.08 (0.00, 0.15) | 0.04 | *Ref.* | -0.05 (-0.22, 0.13) | 0.62 | -0.07 (-0.14, -0.01) | 0.02 | -0.38 (-0.55, -0.20) | < 0.001 |
| **85** | 0.13 (0.00, 0.27) | 0.045 | *Ref.* | 0.03 (-0.34, 0.39) | 0.88 | -0.14 (-0.25, -0.02) | 0.02 | -0.54 (-0.87, -0.20) | 0.002 |
| ^a^Analysis undertaken using joint models (the linear mixed submodel using age as time scale (age, age² and age^3^) is adjusted for sex, marital status, education level and occupation and their interaction with time if it is significant). | | | | | | | | | |
